# Molecular mechanisms involved in fruit cracking: A review

**DOI:** 10.3389/fpls.2023.1130857

**Published:** 2023-03-01

**Authors:** Marlene Santos, Marcos Egea-Cortines, Berta Gonçalves, Manuela Matos

**Affiliations:** ^1^ Department of Genetics and Biotechnology (DGB), University of Trás-os-Montes e Alto Douro (UTAD), Vila Real, Portugal; ^2^ Centre for the Research and Technology of Agro-Environmental and Biological Sciences (CITAB), University of Trás-os-Montes e Alto Douro, Vila Real, Portugal; ^3^ Institute for Innovation, Capacity Building and Sustainability of Agri-food Production (Inov4Agro), University of Trás-os-Montes e Alto Douro, Vila Real, Portugal; ^4^ Instituto de Biotecnología Vegetal, Universidad Politécnica de Cartagena, Cartagena, Spain; ^5^ Department of Biology and Environment (DeBA), University of Trás-os-Montes e Alto Douro, Vila Real, Portugal

**Keywords:** environmental stress, exocarp-specific genes, fruit cracking, gene expression, molecular mechanisms

## Abstract

Several fleshy fruits are highly affected by cracking, a severe physiological disorder that compromises their quality and causes high economical losses to the producers. Cracking can occur due to physiological, genetic or environmental factors and may happen during fruit growth, development and ripening. Moreover, in fleshy fruits, exocarp plays an important role, acting as a mechanical protective barrier, defending against biotic or abiotic factors. Thus, when biochemical properties of the cuticle + epidermis + hypodermis are affected, cracks appear in the fruit skin. The identification of genes involved in development such as cell wall modifications, biosynthesis and transport of cuticular waxes, cuticular membrane deposition and associated transcription factors provides new insights to better understand how fruit cracking is affected by genetic factors. Amongst the major environmental stresses causing cracking are excessive water during fruit development, leading to imbalances in cations such as Ca. This review focus on expression of key genes in these pathways, in their influence in affected fruits and the potential for molecular breeding programs, aiming to develop cultivars more resistant to cracking under adverse environmental conditions.

## Introduction

Fruit cracking is a severe physiological disorder, common to many fruit crops. It affects fruit quality in numerous species of fleshy fruits (Brüggenwirth and Knoche, 2017; Butani et al., 2019; Lara et al., 2019; Schumann et al., 2019; Wang et al., 2021b).These include sweet cherry, plum, apricot, apple, litchi, pomegranate, citrus, banana, avocado, grape, persimmon, peach, tomato, and pistachio (Simon, 2006; Khadivi-Khub, 2015; Correia et al., 2018; Butani et al., 2019). However, sweet cherry, grape and tomato are the crops most affected by cracking due to their susceptibility to damage associated to the large scale of these industries (Brüggenwirth and Knoche, 2017; Schumann et al., 2019). Cracks on the fruit surface reduce the fruit marketability as they cause negative effects in fruit quality such as poor appearance, shelf life diminution and increased susceptibility to infections by fungi and other pathogens causing significant losses in the fresh market (Khadivi-Khub, 2015; Butani et al., 2019; Wang et al., 2021b). So, the cracked fruits can only be used in processing industries (especially for fruit juice) if they aren’t infected by fungi (Simon, 2006; Khadivi-Khub, 2015).

In fruits, the skin, also designed as exocarp, supports the internal cell layers, being considered an essential element in fruits which provides a protective barrier against water loss and pathogen attack (Macnee et al., 2020). The fruit skin comprises three main layers, namely cuticle, epidermis and hypodermis (Knoche and Winkler, 2019). Among them, the hypodermis comprises one to several layers of hypodermal cells while the epidermis is located outside the hypodermis, consisting of in just one cell layer. Epidermis and hypodermis comprise the fruit skin (Knoche and Lang, 2017). The arrangement of hypodermal and epidermal cell layers as well as their thickness highly affect the fruit cracking (Wang et al., 2021b). Outside the epidermis, there is a cuticle or cuticular membrane consisting in lipid polymer that covers the fruit surface, acting as a primary barrier for transport of substances into and out of fruits (Weichert and Knoche, 2006). It also plays an important role in the mechanical properties of the skin (Knoche and Lang, 2017). Thus, the exocarp is a key target in many breeding programs related to cracking (Macnee et al., 2020).

Cracking index (CI) refers to the percentage of cracked fruits in the orchard (Correia et al., 2018). Determining the total number of cracked fruits according the orchards conditions is the most reliable method to access the CI (Christensen, 1972). However, this determination depends on climate conditions as well as the fruit stage development or cultivar, which compromises the method accuracy (Christensen, 1972). Considering the sweet cherry, in the lab, CI can be determined as CI= $\frac{\left(5\text{a }+\;3\text{b }+\text{ c}\right)*100}{250}$ , where a, b and c indicate the number of cracked fruits after 2, 4 and 6h of fruits immersion in distilled water (Christensen, 1972; Correia et al., 2018). Concerning to the position of cracks, there are three main types of fruit cracking: (1) deep cracks in the side of fruits, also called as lateral cracking; (2) small/fine cracks at the fruit apical end, also called as pistillar end and (3) circular or semicircular cracks around the stem end in the cavity region (Simon, 2006; Khadivi-Khub, 2015; Rehman et al., 2015; Correia et al., 2018).

The combination of genetic and environmental factors makes fruit cracking difficult to study, even in controlled conditions. Thus, the basic mechanisms involved in cracking remain unclear. However, researchers have suggested that the high occurrence of fruit cracking can be influenced by several factors, namely physiological, biochemical, environmental, agronomical cultural, anatomical, genetic and postharvest storage factors (Simon, 2006; Khadivi-Khub, 2015; Rehman et al., 2015; Correia et al., 2018; Wang et al., 2021b). Simon (2006) suggested that the species and cultivars susceptibility to cracking is mainly genetic. The two main environmental factors involved in cracking are the quantity of rain and its distribution during the ripening period as well as the soil type (Simon, 2006). Cracking may occur during fruit growth, development and ripening (Khadivi-Khub, 2015). However, it mainly occurs during fruit ripening due to changes in the biochemical properties of the exocarp (Petit et al., 2017; Lara et al., 2019). When fruit tissues are subjected to pressures higher than the mechanical resistance of their cell walls and cuticle, the cracks appear in fruit skin (Brüggenwirth and Knoche, 2017). This mostly occurs when maturation and harvest time coincide with a period of high humidity, causing water movement from the branches and leaves to the fruits due to a large difference in their water potentials (Lara et al., 2014; Petit et al., 2017). Moreover, the combination of high temperatures and low humidity, which make the fruit’s skin hard and inelastic, followed by heavy rains accelerates the growth and expansion of the internal tissues at a faster rate. Once the fruit’s skin remains inelastic and their growth doesn’t coup up with the internal tissues growth, cracks appears in the fruit skin (Butani et al., 2019). This leads to a constant stress supported by the fruit since, in most species, the fruit surface and volume increase during fruit development (Knoche and Lang, 2017). Thus, an uncoordinated internal growth associated to an external environment with high climatic variability results in the appearance of cracks in the fruit surface (Wang et al., 2021b).

Using sweet cherry as a model, cracking is commonly associated adverse environmental conditions. These include rain, wet weather and excessive osmotic water uptake through the fruit surface and skin, fruit peduncle cavity, and also fruit peduncle. Excessive water leads to an increase of flesh turgor, fruit volume and surface. Cracks develop when the limit of extensibility of its skin is exceeded (Weichert and Knoche, 2006; Winkler et al., 2016; Knoche and Lang, 2017). This possible explanation for cracking, known as a critical turgor hypothesis, suggests that fruit peduncle, presence of cracks and cuticle are potential pathways for water uptake in sweet cherry (Knoche and Peschel, 2002; Knoche and Winkler, 2019). Water uptake may occur during and after rainfall when water remains in sweet cherry surface as it is retained in the peduncle cavity and in the stylar end leading to a continuous water uptake after rain (Beyer et al., 2005; Knoche and Winkler, 2019). Another possible explanation for sweet cheery cracking, known as a zipper hypothesis, suggests that a localized skin rupture occurs like a zipper due to a local exposure of skin to water where a succession of events leads to cracks development (Winkler et al., 2016). Strain in the skin during the last stage of fruit growth occurs due to a down regulation of genes involved in wax and cutin biosynthesis, leading to a decrease in cuticle deposition (Alkio et al., 2012; Alkio et al., 2014). A thinner cuticle may not withstand increase of strain in the skin and, consequently, microcracks develop (Winkler et al., 2016).

Fruit cracking in sweet cheery can occur due to several additional factors, because of different cracking susceptibilities of cultivars. These include fruit size and firmness, fruit shape, skin and cuticular properties, osmotic concentration and stomata in the fruit skin, stage of fruit development, and water-retaining capacity of the fruit pulp (Simon, 2006; Balbontín et al., 2013; Khadivi-Khub, 2015; Rehman et al., 2015; Correia et al., 2018). Moreover, Li et al. (2021a) proposed that orchard management like irrigation, growth regulators or mineral applications as well as gene expression related to fruit traits may have a positive relationship with cracking. Simon et al. (2004) reported a positive correlation between cracking and soluble solids content.

### Cuticle as an interface fruit-environment

The cuticle is very important in flesh fruits, as it acts as a mechanical protective barrier against external or internal stresses, either biotic or abiotic, and in defense against pathogens (Petit et al., 2017; Lara et al., 2019; Trivedi et al., 2019). The cuticle is composed of a lipophilic polymer of cutin, waxes, comprising a mixture of very-long-chain fatty acids and their derivatives, and polysaccharides (Knoche and Winkler, 2019; Trivedi et al., 2019). It is a primary barrier in water transport and fruit rot pathogens, responding to environmental conditions like water deficit, changes in relative humidity, temperature or light intensity (Knoche and Winkler, 2019; Lara et al., 2019). It also provides mechanical support for fruit integrity (Zarrouk et al., 2018). So, the cuticle weakening in ripe fruits can cause severe economic losses by developing several visual cuticle-associated traits which are dependent of the interaction among the cuticle and environment and/or the development stage of the fruit (Petit et al., 2017; Lara et al., 2019). Among the several visual cuticle-associated traits can be included fruit color in tomato (Gonzali and Perata, 2021), fruit cracking in sweet cherry (Lane et al., 2000; Simon, 2006; Rehman et al., 2015), tomato (Domínguez et al., 2012), pomegranate (Singh et al., 2020), grape (Sahadev et al., 2017) or litchi (Marboh et al., 2017), brightness in tomato (Petit et al., 2014), russeting in apple (Knoche et al., 2011; Straube et al., 2021) and pear (Zhang et al., 2020; Zhang et al., 2022), and browning in pear (Franck et al., 2007) and litchi (Jiang et al., 2004). The thickness and chemical composition of fruit cuticle is another factor in cuticle-associated traits, presenting a high variability according fruit tree species, cultivars, and fruit development (Knoche and Lang, 2017; Zarrouk et al., 2018). Although the cuticle-associated traits have a considerable phenotypic diversity, they can be linked to genotypic variation (Petit et al., 2017). However, to understand the cuticle-associated traits in crop species, it is essential to identify new cuticle-related genes and the alleles involved in the trait-of-interest to select beneficial cuticle-associated genetic variants for genetic improvement (Petit et al., 2017; Lara et al., 2019). Thus, the identification of genes that play a role in cuticle synthesis and deposition is important to obtain a better knowledge of its function and development (Lara et al., 2014; Lara et al., 2019; Wang et al., 2021b). Identifying genes involved in cuticle development may contribute to develop cracking resistant cultivars by maintaining the cuticle barrier function, and, thus prevent microcracks formation, keep low stomatal density and a thick cuticle (Peschel and Knoche, 2012).

## Molecular mechanisms associated to cracking

Cracking susceptibility of cultivars is considerable among the different species affected by this disorder. Different fruit cultivars present different cracking phenotypes. It is interesting that a cultivar totally tolerant to the disorder has not been described (Balbontín et al., 2013; Butani et al., 2019), this maybe due to a quantitative gene effect based on multiple genes (Khadivi-Khub, 2015; Wang et al., 2021b). So, understanding the genetic factors involved in fruit cracking is essential to select and develop crack-resistant cultivars, which has been one of the major goals in most of the breeding programs (Balbontín et al., 2013; Khadivi-Khub, 2015).

One aim of sweet cherry breeding strategies is to develop more cracking resistant cultivars. Resistance may be associated with genotypes that present low cuticle strain and thick cuticle, maintaining an intact cuticle throughout fruit development. A second type may be genotypes that maintain cutin and wax deposition along fruit growth, especially in the last stage of fruit development (Peschel and Knoche, 2012) Indeed, the characterization of genes related to fruit cuticle development can provide more knowledge about the cuticle functions (Lara et al., 2014). Transcriptomic analyses, shows changes in the expression level of some genes, potentially involved in wax biosynthesis, consistent with wax concentrations (Lara et al., 2019). These include genes related to waxes and cutin biosynthesis and cuticular lipid transporters, whose downregulation leads to a cessation of cuticle deposition (Alkio et al., 2012; Alkio et al., 2014). The cuticular waxes composition varies among fruit species and cultivars (Trivedi et al., 2019).The major plant cuticular waxes components are derived from very-long-chain fatty acids (VLCFAs) and their derivatives like primary and secondary alcohols, alkanes, aldehydes, ketones, and esters (Samuels et al., 2008; Zarrouk et al., 2018; Trivedi et al., 2019). These biomolecules are generated by the *de novo* fatty acid biosynthesis in the plastid followed by fatty acid elongation in the endoplasmic reticulum of the epidermal cells (Zarrouk et al., 2018).

Genes involved in cell wall metabolism affect fruit cracking (Wang et al., 2021b). Cracking rate is influenced by cell wall protopectin and cellulose contents and cell wall thickness (Jiang et al., 2019a). Moreover, the mechanical characteristics of the pericarp, determined by cell wall disassembly, modification, and composition can also contribute to cracking susceptibility (Brüggenwirth and Knoche, 2017). Plant cell wall metabolism regulates the cell wall extensibility, determining cell size and shape (Le Gall et al., 2015). Cell wall degradation and modification has been linked to fruit ripening and softening (Teh et al., 2014). Cell wall-modifying enzymes designated as non-pectolytic enzymes are involved in cell enlargement and expansion by hemicellulose modifications. These include endo-1,4-β-glucanases (EGase), xyloglucan endotransglycosylase/hydrolases (XET/XTH) and expansins (Goulao and Oliveira, 2008; Le Gall et al., 2015). Other cell wall-modifying enzymes, including polygalacturonases (PG), pectin methylesterases (PME), pectin acetylesterases (PAE), pectin/pectate lyases (PL) and *β*-galactosidases (β-Gal), are involved in cell wall plasticity by cleavage or modification of the polysaccharide backbone. Thus, they act as pectolytic enzyme expansins (Goulao and Oliveira, 2008; Le Gall et al., 2015). The properties and structure of cell walls are affected by modifications on the cell wall polysaccharides during ripening (Brummell, 2006). These have been associated to the development of fruit cracking as a result of combined action of cell wall modifying enzymes during fruit ripening and softening (Brummell and Harpster, 2001). Xyloglucan endotransglycosylase is involved in cell wall expansion and re-modelling (Stratilová et al., 2020) by hydrolyzing and re-ligating xyloglucan to other polysaccharides, especially with cellulose. It may control the cell wall relaxation, as the interaction among xyloglucan and cellulose affects plant cells growth control and fruit softening (Kaur, 2019).

Plant growth, both in cell size and number drive fruit expansion and must overcome resistance from the protective cell wall (Marowa et al., 2016). The expansins are involved in cell wall extension acting as regulators of plant cell elongation. Expansins contribute to fruit ripening and softening (Li et al., 2003; Marowa et al., 2016). Expansins, act as zippers to break the hydrogen bonds and unlink cell wall polysaccharides (Marowa et al., 2016). Expansins have been associated with a decrease in cracking index, as they promote the fruit growth by cell walls extensibility and cell expansion (Marowa et al., 2016).

Plant cell wall-modifying enzymes play a key role in fruit ripening, being encoded by multigene families, highlighting their complexity (Brummell, 2006; Goulao and Oliveira, 2008). Moreover, there are increases in expression as well as *de novo* synthesis and activity of cell wall-modifying enzymes, promoting significant modifications in cell wall during ripening (Goulao and Oliveira, 2008).

Transcription factors (TFs) regulate gene expression acting as molecular switches of their target genes binding to different cis-regulatory elements (Franco-Zorrilla et al., 2014; Joshi et al., 2016; Javed et al., 2020). They control all developmental aspects in living cells (Javed et al., 2020). TFs present an important role in plant tolerance/resistance to both biotic and abiotic stresses (Shahzad et al., 2021), by suppressing or activating genes at the transcriptional level (Javed et al., 2020). Some TFs are crucial in biotic and abiotic stresses simultaneously, and also a single TF has the capacity to answer to several stresses (Shahzad et al., 2021). In plants, there are more than 50 TFs families, being WRKY, MYB, NAC (Javed et al., 2020; Shahzad et al., 2021), AP2/ERF (Javed et al., 2020), DREB, bZIP, Zinc-finger, HSF, Dof and NF-Y (Shahzad et al., 2021) the most important involved in biotic and abiotic stresses. So, a better knowledge about TF genes expressed under multiple stresses, may be useful in new crop breeding programs to develop climate-resilient cultivars as well as improve plants yield and health, since an upregulation of TFs is closely related to an increase of tolerance against biotic and abiotic stresses (Shahzad et al., 2021).

**Figure 1:**
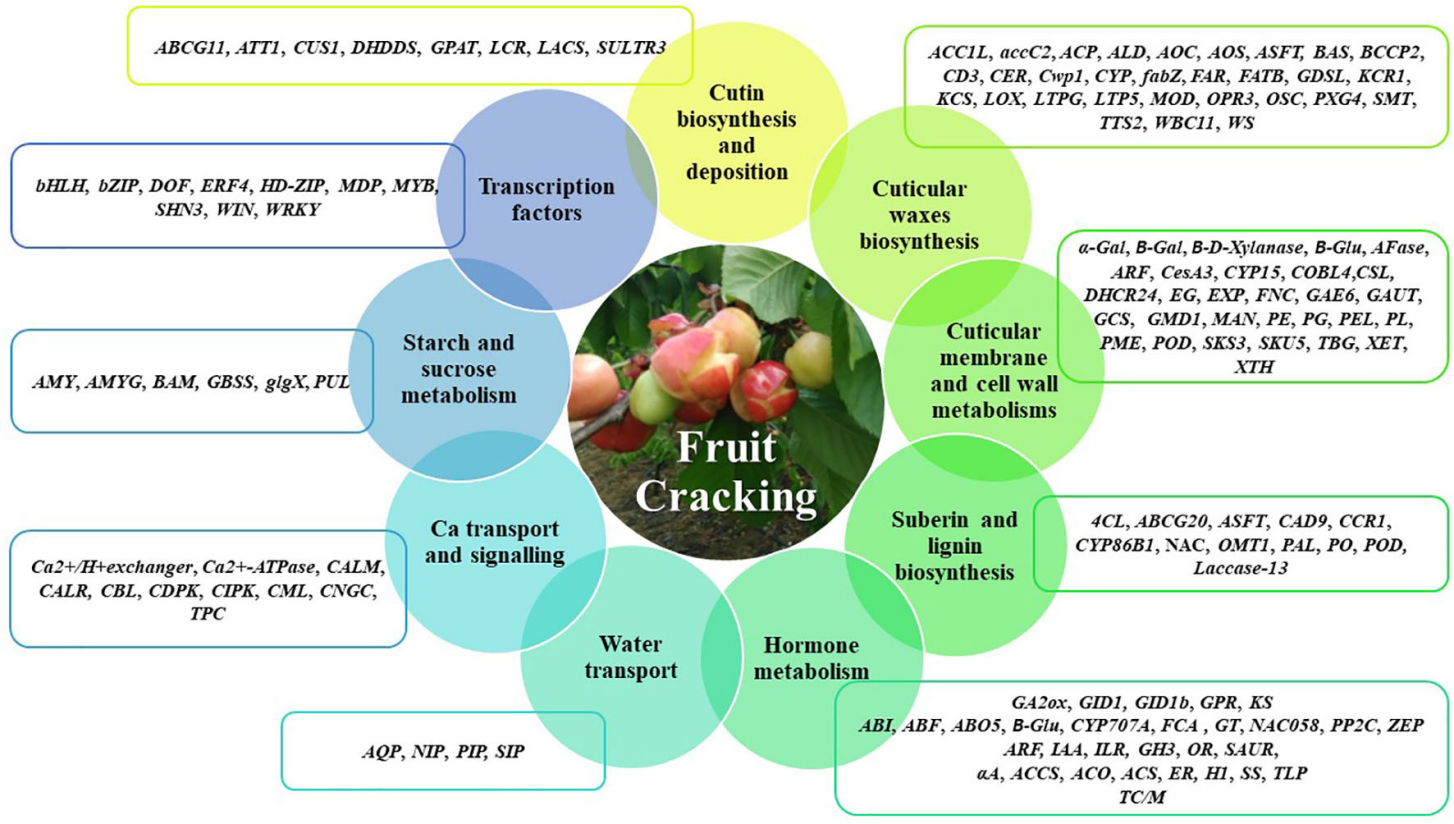
Main gene classes potentially involved in fruit cracking. The genes described belong to the molecular functions of cutin biosynthesis and deposition, cuticular waxes biosynthesis, cuticular membrane and cell wall metabolisms, suberin and lignin biosynthesis, hormone metabolism, water transport, Ca transport and signaling, starch and sucrose metabolisms and transcription factors. Cutin biosynthesis and deposition - *ABCG11 (ATP binding cassette transporter), ATT1 (Cytochrome P450 oxidase CYP86A2), CUS1 (cutin synthase 1), DHDDS (ditrans,polycis-polyprenyl diphosphate synthase), GPAT (Glycerol-3-phosphate acyltransferase), LCR (Cytochrome P450 oxidase CYP86A8), LACS (Long chain fatty acid–CoA synthetase), SULTR3 (sulfate transporter 3)*; Cuticular waxes biosynthesis **-** *ACC1L (acetyl-CoA carboxylase 1-like), accC2 (biotin carboxylase 2, chloroplastic), ACP (Acyl carrier protein), ALD (aminotransferase ALD, chloroplastic-like), AOC (oxide cyclase), AOS (allene oxide synthase), ASFT (Aliphatic suberin feruloyl-transferase), BAS (Beta-amyrin synthase), BCCP2 (biotin carboxyl carrier protein of acetyl-CoA carboxylase 2, chloroplastic), CD3 (cutin deficient 3), CER (Eceriferum family), Cwp1 (cuticular water permeability), CYP (cytochrome-P450 family), fabZ (3-hydroxyacyl-[acyl-carrier-protein] dehydratase FabZ-like), FAR (fatty acyl reductase), FATB (Fatty acyl–ACP-thioesterase B), GDSL (GDSL lípase), KCR1 (b-Ketoacyl-CoA reductase 1), KCS (β-ketoacyl-CoA synthase), LOX (lipoxygenase), LTPG (glycosylphosphatidylinositol-anchored lipid protein), LTP5 (lipid transfer protein 5), MOD (Microsomal oleate desnaturase), OPR3 (12-oxophytodienoate reductase 3), OSC (oxidosqualene cyclase), PXG4 (Putative peroxygenase 4), SMT (24-methylenesterol C-methyltransferase), TTS2 (triterpene synthase 2), WBC11 (ABC-transporter WBC11), WS (wax synthase)*; Cuticular membrane and cell wall metabolisms - *α-Gal (α- galactosidase), B-Gal (β-galactosidase), B-Glu (β-glucosidase), AFase (alpha-L-arabinofuranosidase), ARF (arabinofuranosidase), CesA3 (cellulose synthase), CYP15 (cytochrome P450 monooxygenase/hydrolase), COBL4 (COBRA-like gene 4), CSL (Cellulose synthase-like), DHCR24 (Delta24-sterol reductase), EG (endoglucanase), EXP (Expansin), FNC (Fruit netted-cracking gene), GAE6 (UDP-glucuronate 4-epimerase), GAUT (alpha-1,4-galacturonosyltransferase), GCS (Gamma-glutamylcysteine synthetase, gamma-GCS), GMD1 (GDP-mannose 4,6-dehydratase 1), MAN (Beta-mannanendohydrolase), PE (pectinesterase), PG (polygalacturonase), PEL/PL (pectate lyase), PME (pectin methylesterase), POD (Peroxidase), SKS3 (Pectinesterase-like), SKU5 (Pectinesterase-like), TBG (tomato β-galactosidase), XET (xyloglucan:xyloglucosyl transferase), XTH (Xyloglucan endotransglycosylase/hydrolase)*; Suberin and lignin biosynthesis - *4CL (4-coumaric acid, CoA ligase), ABCG20 (ATP-Binding cassette G20), ASFT (aliphatic suberin feruloyl-transferase), CAD9 (cinnamyl alcohol dehydrogenase9), CCR1 (cinnamoyl CoA reductase), CYP86B1 (Cytochrome P450), NAC (NAC domain containing protein), OMT1 (O-methyltransferase1), PAL (phenylalanine ammonia lyase), PO/POD (Peroxidase)*; Hormone metabolism - *GA2ox (Gibberellin 2-oxidase), GID1 (GA insensitive DWARF1), GPR (Gibberellin-regulated protein), KS (ent-kaurene synthase), ABI (ABA insensitive), ABF (ABRE-binding factors), ABO5 (ABA overly-sensitive 5), B-Glu (β-glucosidase), CYP707A (ABA 8’-hydroxylase), FCA (Flowering time control protein A), GT (ABA glycosyltransferase), NAC058 (ABA signaling gene - NAC Domain containing protein58), PP2C (Protein phosphatase 2C), ZEP (Zeaxanthin epoxidase), ARF (Auxin response Factor), IAA (indole-3-acetic acid), ILR (IAA-amino acid hydrolase), GH3 (IAA-amido synthetase), OR (Oxidoreductase) (NAD+ oxidoreductase), SAUR (Small Auxin Up-Regulated genes), αA (alpha-amylase), ACCS (Aminocyclopropanecarboxylate synthase), ACO (1-aminocyclopropane-1-carboxylic acid oxidase), ACS (1-aminocyclopropane-1-carboxylic acid synthase), ER (Ethylene receptor), H1 (Hydrolase), SS (sucrose synthase), TLP (Thaumatin-like protein), TC/M (terpene cyclase/mutase)*; Water transport - *AQP (Aquaporin), NIP (Aquaporin), PIP (Plasma membrane intrinsic protein), SIP (Aquaporin)*; Ca transport and signalling - *CALM (Calmodulin), CALR (Calreticulin), CBL (calcineurin B-like protein), CDPK (Ca^2+^-dependent protein kinases), CIPK (CBL-interacting serine/threonine-protein kinase), CML (Calmodulin-like protein), CNGC (Cyclic nucleotide-gated ion Channel), TPC (putative voltage-gated Ca^2+^)*; Starch and sucrose metabolism - *AMY (alpha-amylase), AMYG (glucoamylase), BAM (beta-amylase), GBSS (granule-bound starch synthase), glgX (glycogen debranching enzyme), PUL (pullulanase)*; Transcription factors - *bHLH (bHLH transcription factor), bZYP (bZIP transcription factor), DOF (DOF transcription factor), ERF4 (ethylene-responsive transcription factor 4), HD-ZIP (homeobox-leucine zipper protein), MDP (MADS box transcription factor), MYB (MYB domain protein), SHN3 (*ethylene response subfamily member)*, WIN (AP2/EREBP-type transcription factor), WRKY (WRKY family transcription factor)*.

In this context, the review will focus on the main cuticle and cell wall related genes potentially involved in fruit cracking (**Figure 1**). The potentially cracking-related genes in the several fruits highly affected by cracking, such as sweet cherry (*Prunus avium*), apple (*Malus domestica*), watermelon (*Citrullus lanatus*), litchi (*Litchi chinensis*), tomato (*Solanum lycopersicum*), atemoya (*A. cherimola× A. squamosa*), grape (*Vitis vinifera*) and jujube (*Zizyphus jujuba*), are summarized in **Tables 1**–**6** according their putatively role, namely cuticular membrane and cell wall metabolisms (**Table 1**), cutin biosynthesis and deposition (**Table 2**), cuticular waxes biosynthesis (**Table 3**), water transport, calcium transport and signaling, and starch and sucrose metabolism (**Table 4**), fruit hormone metabolisms (**Table 5**) and transcription factors (**Table 6**).

**Table 1:** Potentially cracking-related genes involved in metabolisms of cuticular membrane, cell wall, suberin and lignin biosynthesis.

Genes	Species	Reference
*ABCG20*, *CAD9*, *CCR1*, *CYP86B1*, *NAC038*, *NAC058*, *OMT1*	Apple	Joshi et al. (2018); Straube et al. (2021)
*AFase*, *CesA3*	Grape	Martins et al. (2020); Yu et al. (2020)
*ARF*, *COBL4*, *CSLA9*, *CSL12*, GAE6, *GMD1*, SKS3, *SKU5*	Jujube	Hou et al. (2018)
*ASFT*, *4CL*, *Laccase-13*, *PAL*	Tomato	Zhang et al. (2021b)
*α-GAL*, *AGAL2*, *AGAL1*	Atemoya, Jujube	Hou et al. (2018); Li et al. (2019)
*β-D-Xylanase*	Litchi	Wang et al. (2021a)
*β-Gal (1*, *2*, *5*, *8)*	Apple, Atemoya, Grape, Jujube, Litchi, Sweet Cherry	Kovács et al. (2008); Balbontín et al. (2014); Li et al. (2014); Hou et al. (2018); Joshi et al. (2018); Yang et al. (2018); Li et al. (2019); Correia et al. (2020b); Yu et al. (2020)
*β-Glu*, *BGLU17*	Apple, Atemoya, Grape	Joshi et al. (2018); Chen et al. (2019a); Yu et al. (2020)
*CYP15*	Grape	Martins et al. (2020)
*EG*	Atemoya, Grape, Litchi	Li et al. (2014); Li et al. (2019); Yu et al. (2020); Zhu et al. (2020)
*EXP*, *EXP1*, *EXP2*, *EXP6*, *EXPA3*, *EXPA4*, *EXPA11*, *EXPA15*, *A1.1*, *A8-like*, *A10.2*	Apple, Atemoya, Grape, Jujube, Litchi, Sweet Cherry, Tomato	Brummell et al. (1999); Moctezuma et al. (2003); Yong et al. (2006); Kasai et al. (2008); Balbontín et al. (2014); Li et al. (2014); Hou et al. (2018); Joshi et al. (2018); Jiang et al. (2019a); Li et al. (2019); Correia et al. (2020b); Martins et al. (2020); Xue et al. (2020); Michailidis et al. (2021); Wang et al. (2021a); Zhang et al. (2021b)
*FNC, GCS, TBG4*, *TBG6*	Tomato	Smith et al. (2002); Moctezuma et al. (2003); Xue et al. (2020); Zhang et al. (2021b)
*DHCR24*	Watermelon	Jiang et al. (2019b)
*GAUT*	Atemoya	Chen et al. (2019a)
*MAN*, *MAN5*	Jujube, Tomato	Hou et al. (2018); Xue et al. (2020)
*PE*, *PEL*, *PEL.4*	Atemoya, Grape, Litchi, Sweet Cherry, Tomato	Li et al. (2014); Chen et al. (2019a); Li et al. (2019); Xue et al. (2020); Zhu et al. (2020); Michailidis et al. (2021)
*PG*, *PG1*, *PG2*	Atemoya, Grape, Jujube, Litchi, Tomato	Li et al. (2014); Hou et al. (2018); Chen et al. (2019a); Li et al. (2019); Jiang et al. (2019a); Martins et al. (2020); Xue et al. (2020); Yu et al. (2020); Zhu et al. (2020); Zhang et al. (2021b)
*PL*, *PL1*, *PL2*	Grape, Jujube	Hou et al. (2018); Yu et al. (2020)
*PME*, *PME1*, *PMEI*, *PME3*	Atemoya, Grape, Jujube	Li et al. (2019); Martins et al. (2020); Yu et al. (2020)
*PO1*, *PO2*	Litchi	Wang et al. (2019a)
*POD*, *POD1*, *POD2*	Grape, Tomato, Watermelon	Jiang et al. (2019b); Xue et al. (2020); Zhu et al. (2020)
*XET*, *XET1*, *XET2*, *XET3*	Atemoya, Grape, Litchi, Watermelon	Lu et al. (2006); Li et al. (2014); Jiang et al. (2019b); Li et al. (2019); Zhu et al. (2020)
*XTH*, *XTH7*, *XTH9*	Jujube, Tomato	Hou et al. (2018); Xue et al. (2020)

**Table 2:** Potentially cracking-related genes involved in cutin biosynthesis and deposition.

Genes	Species	Reference
*ABCG11*	Apple	Straube et al. (2021)
*ATT1*	Sweet Cherry	Alkio et al. (2012); Declercq et al. (2014)
*CUS1*	Tomato	Zhang et al. (2021b)
*DHDDS*, *SULTR3*	Watermelon	Jiang et al. (2019b)
*GPAT*	Watermelon	Jiang et al. (2019b)
*GPAT4*	Tomato	Romero and Rose (2019)
*GPAT5*		Zhang et al. (2021b)
*GPAT6*	Apple	Straube et al. (2021)
*GPAT4/8*, *LCR*	Sweet Cherry	Alkio et al. (2012)
*LACS1*	Sweet Cherry	Alkio et al. (2012)
	Tomato	Romero and Rose (2019)
*LACS2*	Sweet Cherry	Alkio et al. (2012); Declercq et al. (2014)

**Table 3:** Potentially cracking-related genes involved in cuticular waxes biosynthesis.

Genes	Species	Reference
*ACC1L*, *accC2*, *BCCP2*, *fabZ*, *PXG4*	Jujube	Li et al. (2020); Li et al. (2021c)
*ACP*, *MOD*	Litchi	Wang et al. (2019a); Wang et al. (2021a)
*ALD1*, *ALD4*, *ALDH3F1*, *AOC*, *FAR2*, *OPR3*	Jujube	Li et al. (2020); Liu et al. (2020)
*AOS*	Jujube	Liu et al. (2020)
	Litchi	Wang et al. (2019a); Wang et al. (2019b)
*ASFT*, *BAS*, *OSC (1*, *3*, *4*, *5)*	Apple	Joshi et al. (2018); Falginella et al. (2021)
*CD3*, *Cwp1*, *FAR*, *TTS2*	Tomato	Hovav et al. (2007); Romero and Rose (2019); Zhang et al. (2021b)
*CER1*	Jujube	Li et al. (2020); Li et al. (2021b)
	Sweet Cherry	Alkio et al. (2012)
*CER3*	Apple	Joshi et al. (2018)
	Jujube	Li et al. (2021b)
	Sweet Cherry	Alkio et al. (2012)
*CER5*	Jujube	Li et al. (2021b)
	Sweet Cherry	Alkio et al. (2012)
*CER6*	Apple	Straube et al. (2021)
	Tomato	Vogg et al. (2004); Romero and Rose (2019)
*CER9*	Grape	Martins et al. (2020)
*CER (4*, *7*, *8*, *14*, *15*, *6*, *18*, *26*, *29)*	Jujube	Li et al. (2021b)
*CER1L1*	Jujube	Li et al. (2021c)
*CYP716A1*	Apple	Joshi et al. (2018)
*CYP (86A*, 86A22, *86B1*,94A2*)*	Jujube	Li et al. (2020); Li et al. (2021c)
*FATB*, *KCR1*, *WBC11*, *WS*	Sweet Cherry	Alkio et al. (2012); Balbontín et al. (2014); Correia et al. (2020b)
*KCS*	Jujube	Liu et al. (2020)
	Tomato	Zhang et al. (2021b)
	Watermelon	Jiang et al. (2019b)
*KCS1*	Jujube	Li et al. (2020)
	Sweet Cherry	Alkio et al. (2012)
*KCS6*	Sweet Cherry	Alkio et al. (2012); Balbontín et al. (2014)
*KCS10*	Apple	Straube et al. (2021)
*KCS12*	Jujube	Li et al. (2020)
*LOX*	Litchi	Wang et al. (2019a)
*LOX2*	Jujube	Liu et al. (2020)
*LTPG1*	Sweet Cherry	Alkio et al. (2012); Balbontín et al. (2014)
*LTPG (2*, *3*, *5*, *6*, *7*, *8*, *11*, *15)*	Apple	Joshi et al. (2018); Gao et al. (2021)
*LTP5*, *GDSL*, *NLTP9*	Tomato	Zhang et al. (2021b)
*SMT*	Watermelon	Jiang et al. (2019b)
*WSD1*	Apple	Straube et al. (2021)

**Table 4:** Potentially cracking-related genes involved in water transport, calcium transport and signaling, and starch and sucrose metabolisms.

	Genes	Species	Reference
**Water Transport**	*AQP*, *NIP*, *SIP*	Litchi	Li et al. (2014)
	*PIP*	Jujube	Ren et al. (2017)
		Litchi	Li et al. (2014); Wang et al. (2021a)
	*PIP1;4*	Sweet Cherry	Breia et al. (2020)
	*PIP2;1*		Michailidis et al. (2021)
	*PIP2A*	Apple	Joshi et al. (2018)
**Calcium transport and signaling**	*Ca^2+^/H^+^exchanger*, *Ca^2+^-ATPase*, *CBL*, *CDPK*, *TPC*	Litchi	Li et al. (2014)
	*CALM*, *CALR*	Jujube	Ren et al. (2017)
	*CIPK*, *CML*, *CNGC*	Litchi	Wang et al. (2021a)
**Starch and sucrose metabolisms**	*AMY*, *AMYG*, *BAM*, *GBSS*, *PUL*	Atemoya	Chen et al. (2019a)
	*glgA*, *glgB*, *glgC*, *glgP*, *glgX*		

**Table 5:** Potentially cracking-related genes involved in fruit hormone metabolisms.

	Genes	Species	Reference
**Gibberellins metabolic pathway**	*GID1b*	Apple	Joshi et al. (2018)
	*GPR*	Litchi	Wang et al. (2021a)
	*GA2ox*, *GID1*, *KS*		Li et al. (2014)
**ABA metabolic pathway**	*ABI*, *ABF2*, *ABF3*, *ABO5*, *FCA*	Sweet Cherry	Michailidis et al. (2021)
	*ABI1*, *ABI5*, *β-Glu*, *GT*	Litchi	Li et al. (2014)
	*CYP707A*		Li et al. (2014); Wang et al. (2019a); Wang et al. (2019b)
	*NAC058*	Apple	Joshi et al. (2018)
	*PP2C*	Litchi	Li et al. (2014); Wang et al. (2021a)
	*ZEP*		Wang et al. (2021a)
**Auxin metabolic pathway**	*ARF*, *IAA*, *ILR*, *SAUR*	Litchi	Wang et al. (2021a)
	*GH3*		Wang et al. (2019a); Wang et al. (2019b); Wang et al. (2021a)
	*OR1*, *OR3*		Wang et al. (2019a); Wang et al. (2019b)
**Ethylene metabolic pathway**	*aA*, *SS*, *TLP*	Litchi	Wang et al. (2019b)
	*ACCS*, *H1*		Wang et al. (2019a); Wang et al. (2019b)
	*ACO*, *ACS*	Litchi	Wang et al. (2021a)
		Sweet Cherry	Michailidis et al. (2021)
	*ER*	Tomato	Xue et al. (2020)
**Brassinosteroid metabolic pathway**	*TC/M*	Litchi	Wang et al. (2019a); Wang et al. (2019b)

**Table 6:** Transcription factors genes potentially involved in fruit cracking.

Genes	Species	Reference
*bHLH*, *bZIP*, *DOF*, *WRKY*	Litchi	Wang et al. (2021a)
*ERF4*	Tomato	Xue et al. (2020)
	Watermelon	Liao et al. (2020)
*HD-ZIP*, *MDP*	Watermelon	Jiang et al. (2019b)
*MYB*	Litchi	Wang et al. (2021a)
*MYB93*, *MYB42*	Apple	Falginella et al. (2021); Straube et al. (2021)
*SHN3*	Apple	Falginella et al. (2021); Straube et al. (2021)
*WINA*, *WINB*	Sweet Cherry	Alkio et al. (2012); Balbontín et al. (2014)

### Cracking-related genes involved in cuticular membrane, cell wall, suberin and lignin biosynthesis

The first work about genes involved in the cuticle formation in sweet cherry was published by Alkio et al. (2012). Based on sequence similarity with *Arabidopsis*, they used the cultivar Regina to identify genes potentially relevant for cuticular membrane (CM) formation. Among the 18 CM target genes identified by Alkio et al. (2012), 15 of them were only detected in the exocarp, meaning that these genes are exocarp-specific. Moreover, 13 of the exocarp-specific genes present a positive correlation with CM deposition, that is, their transcription levels are high when the CM deposition rate is high and low when CM deposition is low. Generally, these genes have higher expression during the first stage of fruit development, when CM deposition is high (Alkio et al., 2012). In sweet cherry, the maximum *β*-galactosidase activity occurs in the early stages of active growth and then decrease abruptly during ripening (Kovács et al., 2008). Similarly, the results provided by Balbontín et al. (2014) also refer that transcript levels of *β-galactosidase* gene vary during fruit development, showing their highest transcript levels in the fruit set stage, declining as ripening advances (**Table 1**). Likewise, Correia et al. (2020b) found different expression levels during fruit development and under different applied compounds, like gibberellic acid, salicylic acid or calcium, in Sweetheart, a cultivar with moderate resistance to cracking. The expansin, *EXP1*, has higher expression in the ripening stage in Kordia, a cracking-resistant cultivar, while in Bing, a cracking-susceptible cultivar, has higher expression in the fruit setting and fruit color change stage (Balbontín et al., 2014). This data has been confirmed by Correia et al. (2020b). There is an increase in expression levels of *EXP1* during fruit development in a cracking-moderate resistant cultivar Sweetheart. Moreover, the expression of the most abundant expansins in sweet cherry (*A1.1*, *A8-like*, *A10.2*) are upregulated in a moderately resistant cultivar Regina compared to the cracking-susceptible cultivar Early Bigi (**Table 1**) (Michailidis et al., 2021). These findings are in agreement with Balbontín et al. (2014) when attested that the more cracking-resistant cultivar present higher gene expression in all stages as well as Correia et al. (2020b) who verified that cherries treated with biostimulant (*Ascophyllum nodosum*) and growth regulators (eg. abscisic acid, glycine betaine or salicylic acid) have lower cracking index, presenting higher transcripts levels of the studied genes, which increase their expression during fruit development. The gene related to pectin metabolism, *PEL.4*, has higher expression levels in the skin of a cracking-susceptible cultivar Early Bigi (Michailidis et al., 2021).

Expansins also play a role in apple fruit development. Wakasa et al. (2003) identified six expansin genes and studied their expression patterns during fruit growth, being *EXPA3* mainly expressed during the fruit enlargement phase. The same was later described by Joshi et al. (2018) for *EXPA4* gene. Moreover, *EXPA3* transcripts in the mesocarp are higher at the fruit color change stage. In the pericarp, *EXPA3* expression is higher at the begin of fruit development and in the ripening stage (Wakasa et al., 2003). This indicates that an accumulation of *EXPA3* mRNA in pericarp reduces the susceptibility of fruit cracking. Early symptoms of fruit cracking coincide with situations in which *EXPA3* gene expression in the mesocarp exceeds the expression in the pericarp (**Table 1**) (Kasai et al., 2008). Expression levels of *β-Gal* genes increase during apple fruit growth and are higher in the mature fruits of cultivar Fuji, a softer and crisper apple, than in fruits of cultivar Qinguan, a firmer and tougher apple (Yang et al., 2018). Among them, *β-Gal1*, *β-Gal2*, and *β-Gal5* genes are highly expressed in fruits, presenting a significant increase of expression patterns until fruit ripening, which suggest that these genes can affect the fruit texture in both types of apple cultivars (Yang et al., 2018). Similarly, there is an upregulation during apples development for *BGAL8* and *BGLU17* cell wall related genes (**Table 1**) (Joshi et al., 2018). Furthermore, an upregulation of genes involved in suberin and lignin synthesis, namely *ABCG20*, *CYP86B1*, *NAC038* and *NAC058*, leads to an increase in suberin content and periderm formation, and thus, to the microcracks development and russet apples (**Table 1**) (Straube et al., 2021). In contrast, an upregulation of the lignin-biosynthesis genes, *CAD9*, *CCR1* and *OMT1* can prevent crack initiation (**Table 1**) (Joshi et al., 2018).

Regarding to suberin and lignin related genes in watermelon, *POD1* gene is upregulated in the cracking-resistant watermelon, while *POD2* is downregulated in cracking-susceptible watermelon. Similarly, the genes involved in cell wall mechanisms, *XET1*, *XET2* and *DHCR24* (**Table 1**) are downregulated in cracking-susceptible watermelon (Jiang et al., 2019b).

Concerning to cell wall related genes in litchi, the analysis of *XET1*, *XET2* and *XET3* genes has different expression patterns among a cracking-resistant cultivar Huaizhi and a cracking-susceptible cultivar Nuomici, but only *XET1* is fruit-specific, once *XET1* transcripts accumulation appeared in pericarp while *XET2* and *XET3* transcripts accumulation enhanced in aril tissues, suggesting that they may play different roles in litchi aril and pericarp growth, and thus, *XET1* is more likely to play a role in reducing litchi fruit cracking than *XET2* and *XET3* (**Table 1**) (Lu et al., 2006). Additionally, the expression of a *XET* gene is upregulated in fruits without cracks compared to cracked fruits (Li et al., 2014). The expression of two genes encoding expansins in litchi pericarp, *Exp1* and *Exp2*, appear to have a closely association with fruit growth and cracking, since the expression of both genes is detected from the early stage of fruit rapid growth and then increase and reach to the highest level at the end of the growth phase in pericarp of the cracking-resistant cultivar Huaizhi, while *Exp1* gene is detected at the stage of rapid fruit growth, and then increase slightly and finally kept almost constant in pericarp of the cracking-susceptible cultivar Nuomici, not being detected expression of *Exp2* in this cultivar (Yong et al., 2006). Similar results were obtained by Wang et al. (2021a), whose an upregulation of cell wall related genes (*EXP* and *β-D-Xylanase*) leads to a low mechanical strength and by Li et al. (2014), who found an upregulation of five *EXP* in fruits without cracks compared to cracked fruits. The same is observed on nine *β-Gal* genes, which are upregulated in fruits without cracks compared to cracked fruits, while five *PG*, one *EG* and three *PE* genes are upregulated in cracked fruits compared to fruits without cracks (**Table 1**) (Li et al., 2014). Regarding to suberin and lignin related genes, an upregulation of *PO1* and *PO2* genes leads to an increase of lignin biosynthesis, resulting in differences in cuticle structure of litchi fruits pericarps with different susceptibilities to cracking compared to cracked pericarps (Wang et al., 2019a).

Bargel and Neinhuis (2005) studied the biomechanics of tomato fruit skin and isolated cuticles, from three cultivars differing in cracking susceptibility and fruit shape, concluding that the cuticle is a mechanically important component of the tomato fruit. Other important contribution to understand the multiple metabolic and genetic phenomena that occur in the fruit skin during ripening was made in tomato by. Mintz-Oron et al. (2008), which describes the differential gene expression at different fruit developmental stages and in different tissues of the fruit and allowed to identify genes expressed specifically in the skin at ripening, such as genes involved in cell wall modification. Also in tomato, a higher expression of *FCN* gene also increase the expression of other genes involved in different metabolic pathways such as suberin metabolism genes (*ASFT* and *GPAT5*), lignin metabolism genes (*4CL*, *PAL* and *Laccase-13*), and cell wall metabolism genes (*EXPA11* and *PG*), which affects the cell wall extensibility, fruit softening, pericarp firmness leading to the appearance of cracks in all fruit surface (**Table 1**) (Zhang et al., 2021b). The simultaneous suppression of *PG* and *EXP1* in ripening fruits reduces cell wall disassembly since *pg/exp* fruits are more firm, present more protopectin and thicker cell walls, concluding that a ripe fruit with more intact pectins in its primary walls is more resistant to cracking (Jiang et al., 2019a). Likewise, antisense inhibition of PE and PG activity affects the level of fruit cracking, while suppression of the ripening related expansin gene (*Exp1*) (Brummell et al., 1999) and tomato *β*-galactosidase 4 (*TBG4*) (Smith et al., 2002) increases fruit firmness. On the other hand, the relationship between activity of cell wall enzymes and cuticular layer was demonstrated in tomato by Moctezuma et al. (2003) as a result of antisense suppression of a *β*-galactosidase gene (*TBG6*), observing a positive correlation between cracked fruit number with low levels of *β*-galactosidase transcripts. The results suggest that the TBG6 product may have an important function in cell wall galactosyl residue metabolism during cell elongation, so the various altered phenotypes observed as a result of *TBG6* gene down-regulation in tomato fruit are further evidence that *β*-galactosidases have important functions in the overall growth and development of tomato fruit (**Table 1**) (Moctezuma et al., 2003). Additionally, the main genes involved in tomato cracking are associated with different metabolic pathways such as cell wall organization, oxidoreductase activity and catalytic activity, which included genes as *MAN*, *PE*, *POD*, *EXP*, *XTH7*, *XTH9*, *PG2*, *ER*, *ERF4* and *gamma-GCS* (Xue et al., 2020). The genes involved in cell wall loosening and expansion, namely *XTH7*, *XTH9*, *PE* and *POD*, are downregulated in a cracking-resistant cultivar and upregulated in a cracking-susceptible cultivar. Likewise, cell-wall degrading enzyme-associated genes are also upregulated, namely *GCS*, *MAN* and *PG* genes as well as ethylene and auxin responsive genes such as *PG*, *PE*, *EXP* and *XTH7* which can be related to cell wall regulation once ethylene influences fruit development and ripening (Xue et al., 2020).

In atemoya, several genes related to cell wall mechanisms were identified, namely 34 *PGs*, 21 *PEs*, 19 *EXPs*, 17 *β-GALs*, 13 *EGs*, 6 *α-GALs*, 6 *PME*, 4 *PELs*, 4 *XETs* and 3 cellulases, which, in general, present higher expression levels in cracked fruits than fruits without cracks, that leads to a reduction in skin elasticity and, consequently, fruits cracking (Li et al., 2019). In addition, *GAUT* gene, involved in pectin synthesis, is upregulated as well as the genes involved in pectin degradation, namely *PE*, *PG* and *PEL*. Likewise, *β*-glucosidase gene, responsible for cellulose degradation, also is upregulated (**Table 1**) (Chen et al., 2019a).

The effect of calcium in genes involved in cell modifications was studied by Yu et al. (2020) using a cracking-susceptible cultivar Xiangfei, whose expression was analyzed in grape berry skins after 1, 2 and 3 weeks of the calcium applications comparing to fruits without calcium application (as a control). Among then, five polygalacturonases (*PG*) and three endoglucanases (*EG*) were studied, showing a downregulation of almost all *PG* genes by calcium at all weeks as well as a downregulation of two *EG* genes in the first and second weeks, and then an upregulation of these genes in the third week (**Table 1**) (Yu et al., 2020). Similar results were obtained by Zhu et al. (2020) using grapes of the same cultivar, Xiangfei, during the ripening stage, finding an upregulation of two *EGs* and one *PG* along the fruit maturation. Other genes involved in cell modifications namely one pectin methylesterase (*PME1*), two polygalacturonases (*PG1* and *PG2*), one expansin (*EXP6*) and one cellulose synthase (*CesA3*) as well as a cuticle biosynthesis gene, cytochrome P450 monooxygenase/hydrolase (*CYP15*), were studied at pulp and skin of grape berry, also with and without calcium treatment in the cultivar Vinhão, showing a downregulation of the genes *PME1*, *PG1*, *PG2*, *EXP6* and *CYP15* promoted by calcium in skin and pulp, while *CesA3* gene is not significantly affected by calcium in the skin and pulp (**Table 1**) (Martins et al., 2020). This suggest a regulation by calcium at transcriptional level and also that calcium can inhibit additional enzymatic pathways involved in cell wall mechanisms (Martins et al., 2020). The work performed by Yu et al. (2020) also analyzed four pectate lyases (*PL*), ten pectin methylesterase (*PME*), two PME inhibitors (*PMEI*), one alpha-L-arabinofuranosidase (*AFase*), four *β*-galactosidase (*β-Gal*), and one *β*-glucosidase (*β-Glu*), all involved in cell wall modifications, in grape berry skins after 1, 2 and 3 weeks of the calcium applications. Among the analyzed genes, the *AFase*, the *β-Glu*, almost all *β-Gal* genes and one *PME* are downregulated by calcium at all weeks, while two *PME* genes are downregulated in the first week after calcium application, four *PME* genes are downregulated in the second week and three *PME* genes are downregulated in the third week (Yu et al., 2020). Regarding *PL* genes, two are downregulated by calcium at first and second weeks and upregulated at third week, while the other two *PL* genes are upregulated during the three weeks. Moreover, there is also one *PMEI* gene continuously and significantly upregulated by calcium and another *PMEI* gene downregulated in the first week (**Table 1**) (Yu et al., 2020). Based in the results, calcium applications in grape berry appear to induce specific modifications both in skin and in pulp, inhibiting pectin degradation and cell wall loosening, and changing the cuticle structure (Martins et al., 2020) as well as leading to cell wall disassembly inhibition, and promoting cell wall strengthening (Yu et al., 2020), playing an important role in preventing cracking. In order to analyze the transcriptome and identify important metabolisms related to grape berry cracking, Zhu et al. (2020) made a RNA-Seq analysis to assess the expression of pericarp genes during the ripening stage, namely at 1 (W1), 2 (W2) and 3 (W3) weeks after veraison in cultivar Xiangfei. The three groups (W1, W2 and W3) presented a similar number of expressed genes, however with different expression in each group (some genes express in all groups, but some genes only express in one unique group) (Zhang et al., 2021a). Comparing W1, W2 and W3, the authors detected an increase of cracking during repining highlighting great changes in gene expression during this period, which can be correlated with 303 DEGs up-regulated and 354 DEGs down-regulated in W2 and W3 relatively to W1 (Zhu et al., 2020). During the fruit development, the cell wall mechanical properties have an important role (Brüggenwirth and Knoche, 2017), so Zhu et al. (2020) validated the RNA-Seq results with a qRT-PCR analysis of genes involved in cell mechanisms, obtaining highly consistent results for both analysis. Among the DEGs involved in cell wall mechanisms, one peroxidase (*POD*), one pectinesterase (*PE*) and eleven xyloglucan endotransglycosylase (*XET*), beyond two *EGs* and one *PG*, were identified (**Table 1**). These genes have differential expression when W2 and W3 groups are compared to W1, showing an up-regulated expression along fruit maturation, which suggested that cell wall related genes play important roles in grape berry cracking regulation (Zhu et al., 2020).

By a transcriptomic analysis of jujube using young and mature fruit, Hou et al. (2018) found several differentially expressed genes, 19 of them related to cell wall mechanisms and fruit ripening. The analysis of gene expression revealed that *AGAL2*, *BGAL*, *EXP2*, *EXPA15*, *PL2*, *SKU5*, *GAE6* and *XTH* genes are upregulated in young fruit stage while *ARF*, *CSL12*, *CSLA9*, *COBL4*, *GMD1*, *PL1* and *PG1* genes are upregulated in ripe fruit stage. However, *AGAL1*, *MAN5*, *PME3* and *SKS3* genes, don’t present significant expression differences among the two ripening fruit stages (**Table 1**) (Hou et al., 2018). Thus, these differences in gene expression during fruit development leads to important changes in cell wall metabolisms playing important roles in preventing or enhancing jujube cracking (Hou et al., 2018).

### Cracking-related genes involved in cutin biosynthesis and deposition, and in cuticular waxes biosynthesis

In sweet cherry, the expression of genes involved in cutin biosynthesis and deposition, namely *LCR* and *LACS1* genes, is detected in the beginning of fruit development, while expression of *ATT1* and *LACS2* (**Table 2**) have higher expression at later fruit stages (Alkio et al., 2012). The previous data was confirmed by Declercq et al. (2014) for *LACS2* and *ATT1* genes, concluding that the expression of these genes increase cutin deposition and decrease cuticle permeability. They may be involved in the molecular mechanisms of sweet cherry cracking. Additionally, expression of *GPAT4/8* is detected in the beginning of fruit development and increase again in the final phase of fruit development (Alkio et al., 2012). Concerning cuticular waxes related genes, more specifically involved in biosynthesis of VLCFAS, *KCS6* and *KCS1* present higher expression in the beginning of fruit development and increased again in the final phase of fruit development as well as the expression of *Lipase* (**Table 3**) (Alkio et al., 2012). Likewise, Balbontín et al. (2014) found higher expression of *KCS6* at fruit setting (begin of fruit development) and fruit color change stage in a cracking-susceptible cultivar Bing, and higher expression in a cracking-resistant cultivar Kordia, coinciding with the ripening of fruits. Additionally, the wax synthase gene, *WS*, presents similar expression levels to *KCS6* (Balbontín et al., 2014). These results have been again corroborated by Correia et al. (2020b) analyzing the expression patterns of *WS* gene in a cracking-moderate resistant cultivar Sweetheart. *WS* increases during fruit development, leading to higher wax content (Correia et al., 2020b). The genes involved in lipid transport, *LTPG1*, *WBC11* and *CER1* show higher expression in the begin of fruit development and, again, in the fruit ripening stage (**Table 3**) (Alkio et al., 2012). The expression of *KCR1* and *FATB* genes is higher in the exocarp in the beginning of fruit development and again in the final fruit development phase. The expression of *CER5* and *CER3* genes is also exocarp-specific at all developmental stages but do not correlate with the CM deposition rate (Alkio et al., 2012).

In apples, the presence of microcracks can lead to russeting (rough and brownish patches on the fruit skin) which can occur due to water uptake on the fruit’s surface (Khadivi-Khub, 2015; Straube et al., 2021). Straube et al. (2021) studied the effect of moisture exposure in apples verifying a decrease in cutin and wax contents due to a down-regulation of genes involved in cutin and wax synthesis (*ABCG11*, *GPAT6*, *KCS10*, *WSD1* and *CER6* genes) (**Table 2** and **Table 3**). This leads to a decrease in cuticle formation and, consequently, to microcracks formation. The *LTPG* genes codify proteins involved in lipid transport. Studies performed by Joshi et al. (2018) show a significant upregulation of *LTPG5* gene as well as of *CER3* and *ASFT* during apple fruit development. The expression of *LTPG* genes is responsive to abiotic stresses and stress hormones such as drought, cold, salt, salicylic acid and jasmonate (Gao et al., 2021). Among 26 potential *LTPG* genes, 9 of them are highly expressed in fruits, including *LTPG2*, *LTPG3*, *LTPG5*, *LTPG6*, *LTPG7*, *LTPG8*, *LTPG11*, *LTPG15*, and *LTPG19*, highlighting the function of this gene family in wax biosynthesis and cracking prevention (**Table 3**) (Gao et al., 2021). Triterpenes are components of surface waxes (Thimmappa et al., 2014). There is a close relationship between the expression of oxidosqualene cyclase (*OSC*) genes and russeting level in apples. *OSC1* and *OSC3* genes present low expression in cultivar Rugiada that generally shows fully russeted skin. In contrast, *OSC1* and *OSC3* are highly expressed in cultivars Smoothee and Golden Delicious with low and moderate russeting, respectively (Falginella et al., 2021). *OSC4* and *OSC5* genes are downregulated in cultivars Smoothee and Golden Delicious and upregulated in Rugiada, correlating with high russeting level in this cultivar (**Table 3**) (Falginella et al., 2021). Likewise, the *β*-amyrin biosynthesis-related genes, *BAS* and *CYP716A1*, are upregulated during fruit development (Joshi et al., 2018).

The watermelon rind has an important role in fruit cracking, being the rind hardness positively correlated with cracking resistance of this fruit (Liao et al., 2020). The expression of genes related to cracking was studied in watermelon (*Citrullus lanatus*), namely cutin related genes, *GPAT*, *DHDDS* and *SULTR3* (**Table 2**) and genes involved in cuticular waxes biosynthesis, *SMT* and *KCS* (**Table 3**), using a cracking-resistant and a cracking-susceptible watermelon cultivars (Jiang et al., 2019b). The results show that the *DHDDS* and *SULTR3* genes are upregulated in the cracking-resistant watermelon, while *GPAT*, *SMT* and *KCS* genes are downregulated in cracking-susceptible watermelon (Jiang et al., 2019b).

In litchi (*Litchi chinensis*), the pericarps of fruits without cracks from cultivars with different cracking susceptibilities and cracked fruits present differences in cuticle structure as a result of an upregulation of genes related to fatty acids such as *LOX*, *MOD* and *AOS* (**Table 3**) (Wang et al., 2019a) as well as a downregulation of lipid synthesis genes, like *ACP*, which is responsible by a low pericarp mechanical strength (Wang et al., 2021a). Moreover, the upregulation of *AOS* gene is found in cracked fruits (Wang et al., 2019b).

The expression of genes related to cutin in tomato, like *LACS1* (long-chain acyl-CoA synthase 1), *CUS1* (cutin synthase) and *GPAT4* (glycerol-3-phosphate acyltransferase 4), decreases during the fruit development and is higher in fruits under water stress, being consistent with the developmental regulation of cuticle (**Table 2**) (Romero and Rose, 2019). Further evidence of the relationship between fruit cracking and properties and composition of the cuticle in tomato is provided by Vogg et al. (2004), where the mutation of the *CER6* gene (*β*-ketoacyl-CoA synthase) leads to an alteration of the cuticular wax composition and increases water permeability (Hovav et al., 2007). Additionally, the genes *TTS2* (triterpene synthase 2) and *CD3* (cutin deficient 3) beyond *CER6* (eceriferum 6), all involved in wax biosynthesis, transport, deposition and regulation, decrease their expression during the fruit development and are higher in fruits under water stress, suggesting that water availability affects the cuticle properties (**Table 3**) (Romero and Rose, 2019). Moreover, the *Cwp1* gene (cuticular water permeability) when expressed leads to fruit dehydration and consequently causes microcracks in tomato cuticle (Hovav et al., 2007). The higher expression of *FNC* gene is responsible by cracking all over the tomato pericarp, whose expression affects other cracking related genes, namely by increasing the expression of genes involved in lipid metabolism such as *GDSL*, *KCS* and *FAR*, and lipid transport like *NLTP9* and *LTP5*, affecting the cuticle elasticity and wax content and thud, cracks appear in fruit surface (**Table 3**) (Zhang et al., 2021b).

Concerning to genes related to wax formation in grape, Martins et al. (2020) verified a downregulation of one E3 ubiquitin ligase (*CER9*) when calcium was applied in the cultivar Vinhão (**Table 3**).

In last years, several studies related to biosynthesis of cuticular waxes and related genes in jujube have been developed (Li et al., 2020; Liu et al., 2020; Li et al., 2021b; Li et al., 2021c). A RNA-Seq analysis, using fruits with and without cracking, to analyze the genes differentially expressed in cracked and non-cracked jujube fruits, allowed to find 785 up-regulated and 251 down-regulated genes in cracked fruits, which are involved in several metabolic processes, namely surface wax production in cracked fruits (Liu et al., 2020). The expression of genes involved in fatty acid biosynthesis (*FAR2*) and fatty acid elongation (*KCS1* and *KCS12*) was studied in jujube fruits at different maturation stages, namely white-ripe, coloring, and full-red development stages, collected from cultivars with different cracking susceptibilities (a highly cracking-resistant cultivar Popozao, a cracking-resistant cultivar Banzao, and a cracking-susceptible cultivar Hupingzao), showing higher expression for *FAR2* and *KCS12* genes in coloring stage, being higher in the more resistant cultivar, and lower in the more susceptible cultivar while *KCS1* gene presents similar expression during fruit development and for all cultivars (Li et al., 2020). Moreover, the 3-ketoacyl-CoA synthase (*KCS*) gene, related to the synthetic pathway of cutin, is highly upregulated in cracked fruits, which leads to alterations in biosynthesis in cuticle wax and, consequently, jujube cracking (**Table 3**) (Liu et al., 2020). Furthermore, Li et al. (2020) also analyzed the genes involved in fatty acid degradation, namely *ALD1*, *ALD4* and *ALDH3F1* genes, whose expression of *ALD1* and *ALDH3F1* genes is upregulated in coloring stage in cracking-resistant cultivar while the expression of *ALD4* gene is upregulated in cracking-susceptible cultivar at the same stage (**Table 3**). Likewise, a transcriptomic analysis carried out by Li et al. (2021c) to access the wax metabolism pathways in jujube using RNA from fruit pericarp at different maturation stages of a cracking-resistant cultivar and a cracking-susceptible cultivar allowed to identify different metabolic pathways related to wax metabolism, namely fatty acid biosynthesis, fatty acid metabolism and cutin, suberin and wax biosynthesis. All identified genes, in general, increase their expression during fruit development; however, genes *CER1L1* (eceriferum 1-like), *BCCP2* (biotin carboxyl carrier protein of acetyl-CoA carboxylase 2, chloroplastic), *ACC1L* (acetyl-CoA carboxylase 1-like) and *CYP86A22* (cytochrome-P450 86A22) have more expression in cracking-resistant cultivar, while genes *accC2* (biotin carboxylase 2, chloroplastic) and *fabZ* (3-hydroxyacyl-[acyl-carrier-protein] dehydratase FabZ-like) present higher expression in cracking-susceptible cultivar (**Table 3**) (Li et al., 2021c). Concerning to genes involved in jujube wax biosynthesis (*CYP94A2*, *PXG4*, *CYP86A*, *CER1* and *CYP86B1*), in general, are higher in white-ripe period, however *PXG4*, *CYP86A*, *CER1* and *CYP86B1* genes maintain higher expression in the cracking-resistant cultivar than in the other cultivars in coloring stage (**Table 3**) (Li et al., 2020). *CER* genes represent a family of genes with an important role in waxes biosynthesis in jujube, being identified 29 candidate genes (named *CER1* to *CER29*), twelve of them present differences in expression among two cultivars with different susceptibilities to cracking (Li et al., 2021b). Among them, *CER7*, *CER14*, *CER15* and *CER16* genes have higher expression in cracking-susceptible cultivar, while *CER29* has more expression in cracking-resistant cultivar. In addition, *CER26* only has expression in cracking-resistant cultivar (**Table 3**) (Li et al., 2021b). In the biosynthesis of cuticular waxes, in general, the highly cracking-resistant cultivar present higher gene expression in coloring period while cracking-susceptible cultivars have higher expression in white-ripe, decreasing during fruit development and, thus, during the wax formation, the cracking-resistant cultivar synthesize more very-long-chain alkanes and aldehydes, accompanying the fruit surface enlargement, which reduces the jujube cracking (Li et al., 2020). The biosynthesis of jasmonic acid (JA) associated with α-linolenic metabolism (as the precursor of JA) is the main associated to cracking in which the allene oxide cyclase (*AOC*), allene oxide synthase (*AOS*) and 12-oxophytodienoate reductase 3 (*OPR3*) genes are upregulated, while lipoxygenase 2 (*LOX2*) gene is downregulated in cracked jujube fruits when compared with non-cracked jujube fruits (**Table 3**) (Liu et al., 2020).

### Cracking-related genes involved in water transport, calcium transport and signaling, and starch and sucrose metabolisms

Aquaporins (AQPs) transport water, and water uptake is associated to rain-induced cracking in sweet cherries. A better knowledge about these proteins in exocarp and the involvement of AQPs in water penetration through microcracks as well as the transcriptional profile of the genes that codify AQPs can provide new finds about sweet cherry cracking (Chen et al., 2019b). Chen et al. (2019b) identified 25 putative aquaporins genes in sweet cherry, 16 of them express in fruit. An example of the aquaporin role in sweet cherry cracking was provided by Michailidis et al. (2021), who verified that the expression of aquaporin gene, *PIP2;1*, is upregulated in the skin of Early Bigi cultivar (**Table 4**). Moreover, Breia et al. (2020) found an upregulation of *PIP1;4* gene under pre-harvest application of CaCl_2_ in cultivar Skeena, suggesting that this AQP is involved in water transport and, possibly, in crack prevention. Likewise, the aquaporin gene *PIP2A* (**Table 4**) is upregulated during apple development, which may prevent crack initiation (Joshi et al., 2018).

By a high-throughput RNA sequencing (RNA-Seq), the transcriptome of litchi pericarp revealed four genes (*AQP*, 1; *PIP*, 1; *NIP*, 1; *SIP*, 1) involved in water transport and 13 genes (*TPC*, 1; *Ca^2+^/H^+^ exchanger*, 3; *Ca^2+^-ATPase*, 4; *CDPK*, 2; *CBL*, 3) involved in Ca transport (**Table 4**), whose expression present significant differences among cracked fruits and fruits without cracks (Li et al., 2014). Furthermore, a downregulation of calcium transport and signaling genes, like *CIPK*, *CML* and *CNGC* provoke a decrease of the mechanical strength of pericarp of a cracking-susceptible cultivar Nuomici (Wang et al., 2021a).

The genes involved in starch and sucrose metabolism pathways also appear to have an important role in atemoya cracking, since genes involved in starch synthesis such as *glgA*, *glgB*, *glgC* and *GBSS* are mainly downregulated in cracked fruits, while genes involved in starch degradation like *AMYG*, *PUL*, *AMY*, *BAM*, *glgX* and *glgP* are mainly upregulated in cracked fruits (**Table 4**) (Chen et al., 2019a).

By a transcriptomic analysis, a set of 12 cracking-resistant fruits and 12 cracking-susceptible fruits were analyzed to study the gene expression in both types of fruits, finding 218 upregulated genes and 173 downregulated genes, being the aquaporin *PIP* (involved in water absorption), *CALM* and *CALR* (both involved in calcium transport and regulation) genes (**Table 4**), the most related to jujube cracking (Ren et al., 2017). All these genes have higher expression in cracking-resistant fruits than in cracking-susceptible fruits, playing an important role in preventing cracking (Ren et al., 2017).

### Cracking-related genes involved in fruit hormone metabolism

In sweet cherry, the *ABF2*, *ABF3*, *ABO5*, *ABI* and *FCA* genes, involved in abscisic acid metabolism, are downregulated in the cracking-susceptible-cultivar Early Bigi, while *ACS* and *ACO* genes, involved in ethylene biosynthesis, are upregulated in a cracking-moderate resistant cultivar Regina (**Table 5**) (Michailidis et al., 2021). As these plant growth regulators are directly involved in abiotic stress signaling they may pose a mechanistic mode of action to understand the environmental effects on fruit cracking in cherry.

During apple fruit development, the ABA signaling gene, *NAC058* (**Table 5**), is upregulated, and may prevent the cracking initiation (Joshi et al., 2018).

In watermelon, *CHDH* and *GST* genes (**Table 5**) are possibly related to hormone metabolism, whose expression of *GST* gene is upregulated in the cracking-resistant watermelon, while *CHDH* gene is downregulated in cracking-susceptible watermelon (Jiang et al., 2019b).

A balance among pericarp strength and aril expanding pressure can be responsible by litchi cracking, which can occur due an unbalance of plant hormone metabolisms (Wang et al., 2019b; Wang et al., 2021a). Different expression levels of genes related to hormone metabolism was described by Li et al. (2014), namely in five genes (*KS*, 2; *GA2ox*, 2; *GID1*, 1) involved in GA metabolism and 21 genes (*CYP707A*, 2; *GT*, 9; *β-Glu*, 6; *PP2C*, 2; *ABI1*, 1; *ABI5*, 1) involved in ABA metabolism in fruits with and without cracks (**Table 5**). Moreover, genes related to auxins (*GH3*, *IAA* and *ARF*), gibberellins (*GPRs*), and ethylene (*ACO* and *ACS*) are downregulated in litchi pericarp, while genes involved in auxin metabolism (*ILR*, *SAUR* and *ARF*) and ABA metabolism (*ZEP* and *PP2C*) are upregulated in litchi aril, leading to differences in fruit development and pericarp mechanical strength (**Table 5**) (Wang et al., 2021a). Using the transcriptomic and metabolomics analysis, Wang et al. (2019a); Wang et al. (2019b) and Wang et al. (2021a) suggested that the susceptibility to litchi cracking may be associated with the difference in hormone balance of the two analyzed cultivars, that is, differences in metabolism of IAA (indoleacetic acid, natural auxin), ABA (abscisic acid), ethylene, BR (brassinosteroid) and JA (jasmonic acid) once the different metabolites generated by different hormone metabolism are cultivar specific and have distinct expression patterns in the three types of litchi pericarps. Thus, changes in gene expression and metabolites can explain the cracking susceptibility, namely a downregulation of *OR1* and *OR3* genes, and *ACCS* and *H1* genes, involved in IAA and ethylene metabolisms, respectively, and an upregulation of *CYP707A* and *TC/M* genes involved in ABA and BR metabolisms, respectively, in cracking-resistant cultivar Feizixiao; in contrast, *AOS* (BR metabolism) gene present highest expression in cracked fruits from cracking-susceptible cultivar Baitangying (**Table 5**) (Wang et al., 2019a; Wang et al., 2019b). Also, in the ethylene metabolism associated to sucrose synthesis and sweetness increase, *aA* and *SS* genes are downregulated in a cracking-resistant cultivar while *TLP* gene is upregulated in cracked fruits from cracking-susceptible cultivar (Wang et al., 2019a; Wang et al., 2019b). Additionally, *GH3* gene, involved in IAA, is upregulated in cracking-resistant cultivar (Wang et al., 2019a; Wang et al., 2019b; Wang et al., 2021a).

Cracking in atemoya seems to have a close relation with phytohormones, so, several hormones related genes has been identified. The majority is related to auxin and ABA pathways, with 18 and 12 genes, respectively, while cytokinin pathway presented 6 genes, salicylic acid pathway comprised 5 genes, gibberellin and jasmonic acid pathways included 3 genes and ethylene pathway only presented one gene. Comparing with fruits without cracks, the auxin and jasmonic acid related genes were downregulated in cracked fruits, while ABA, cytokinin, gibberellin, ethylene and salicylic acid related genes were upregulated, showing that these genes can have an important role in cracking (Li et al., 2019).

### Transcription factors genes involved in fruit cracking

It’s known that different final products can be generated due to differential regulation by transcription factors according to environmental or development stimuli (Falginella et al., 2021).

In sweet cherry, *WINA* and *WINB* genes encode AP2/EREBP-type transcription factors, regulating several genes involved in cutin and wax biosynthesis (**Table 6**) (Alkio et al., 2012; Balbontín et al., 2014). According to Alkio et al. (2012), these genes present higher expression in the initial stage of fruit development. Balbontín et al. (2014) obtained similar results for *WINB*, finding higher expression levels in the beginning of fruit development in a cracking-resistant cultivar Kordia. This indicates that *WINB* can influence the expression of other cuticular waxes related genes during the fruit growth.

Likewise, a down-regulation of *SHN3* leads to low cutin and wax contents and, consequently, to microcracks development in apple (**Table 6**) (Straube et al., 2021). *SHN3* expression compromises cuticle formation, being considered an essential regulator of apple cuticle biosynthesis (Lashbrooke et al., 2015). Similar results were obtained by Falginella et al. (2021) in which high levels of *SHN3*, are related to high cutin and wax contents, and low russet development, while low levels of *SHN3* transcripts leads to a decrease in cutin and wax contents as well as microcracks development and consequently, high russet development. In contrast, a lower expression of transcription factor *MYB93*, related to suberin and lignin synthesis, leads to low suberin content, resulting in low russet development, while a higher gene expression promotes an increase in suberin, allowing the microcracks development and, consequently, high russet development (Falginella et al., 2021) as also described by Straube et al. (2021) for the *MYB93* and *MYB42* genes (**Table 6**).

The ethylene-responsive transcription factor 4, *ERF4*, is considered the major gene underlying watermelon rind hardness regulation and thus an important factor in cracking resistance. However, beyond the function of *ERF4* in rind hardness variability and consequently in cracking resistance, their expression can be affected by the regulation of other genes such as genes involved in cell wall modification and/or degradation as well as genes related to lignin biosynthesis (Liao et al., 2020). Moreover, *MDP* gene is upregulated in the cracking-resistant watermelon, while the expression level of *HD-ZIP* in cracking-resistant watermelon is lower than in cracking-susceptible watermelon (Jiang et al., 2019b).

In litchi, a decrease of the pericarp mechanical strength can occur due a downregulation of transcription factor genes, such as *WRKY*, *bZIP*, *bHLH* and *MYB* causing a development retardation in fruit and (**Table 6**) (Wang et al., 2021a). In contrast, an upregulation of transcription factor genes, like *WRKY*, *bHLH*, *DOF* and *MYB* cause an upregulation of starch/sucrose metabolism related genes and sugar/water transport and, thus, differences in mechanical strength of pericarp (Wang et al., 2021a).

### Other potential genes/pathways involved in fruit cracking

During apple development, Joshi et al. (2018) found a significant upregulation in the majority of the genes during fruit development, suggesting that a high expression of cuticle-related genes can prevent crack initiation and possibly enhancing cuticular cracking repair.

Atemoya cracking can occur due to an unbalance among genes involved in hormone metabolisms (Li et al., 2019) as well as genes involved in starch metabolism and genes related to cell wall, whereby the transformation of starch into soluble sugars leads to an increase in turgor pressure and, consequently, in cells and tissues rupture. At the same time, the pectin and cellulose degradation decreases cell wall toughness, which together with starch metabolism leads to cracking development in the fruit pericarp (Chen et al., 2019a).

Furthermore, Zhang et al. (2021a) carried out an enrichment analysis during veraison and maturity stages using grapes that were treated with calcium as cracking mitigation strategy, to analyze the transcriptome and secondary metabolites in grape berry cracking. The enrichment analysis under the application of calcium sprays revealed that the main DEGs are related to flavone and flavonol biosynthesis pathway and also to flavonoid metabolism pathway, meaning that the balance of up and down regulation of genes involved in both pathways determine the grape berry cracking rate, and thus, allowing to identify new pathways and genes involved in grape berry cracking and also explain the role of calcium sprays in modulating these pathways and their effect in reducing cracking rate (Zhang et al., 2021a). However, Zhang et al. (2021a) suggest that other metabolic pathways can be involved in grape berry cracking.

In jujube, Hou et al. (2022) used cracked and non-cracked fruits of two cracking-susceptible cultivars, and non-cracked fruits of a cracking-resistant cultivar to elucidate cracking-related molecular mechanisms. Comparing samples from the cracking-resistant cultivar to samples with and without cracking of each cracking-susceptible cultivar, the authors found several cracking related genes which are involved in different metabolic pathways, namely in water transport, cell wall metabolism, starch and sucrose metabolism, cuticle structure, calcium transport, ABA metabolism, indoleacetic acid metabolism, jasmonic acid metabolism, gibberellic acid metabolism and transcription factors (Hou et al., 2022). In general, all cracking related genes involved in these pathways are upregulated in cracked fruits, compared to the non-cracked fruits and, thus, the authors propose that the high expression levels in cracked fruits leads to an increase in the turgor pressure and a decrease in the exocarp mechanical strength, which can lead to the fruit cracking development (Hou et al., 2022).

## Importance for new molecular breeding

Genomics with the development of complete reference genomes, allows to find interesting molecular opportunities for the identification of candidate genes linked to agronomic traits (Bianchi et al., 2015; Soundararajan et al., 2019). Moreover, by the development of omics, such as metabolomics and transcriptomics, associated with whole-genome sequences provides great information about the molecular mechanisms in fruits (Zhang and Hao, 2020; Wang et al., 2021b). Thus, the combination of genomic, transcriptomic and metabolomic analyses can reveal important knowledge and genetic basis for crop’s molecular breeding (Zhang and Hao, 2020). Although the gene expression can be affected by internal and external factors, by the combined information provided by different omics, it is possible to identify transcription factors and key genes of different biosynthesis pathways as well as to understand how environmental conditions affects the traits of interest at molecular level, and consequently improve fruit quality and molecular breeding programs (García-Gómez et al., 2020).

Fruit trees are economically important, but the lengthy life cycles of several years slow the study at the genetic level. However, molecular tools represent a good strategy to understand adaptation to abiotic stresses and environmental conditions (Licciardello et al., 2021). So, it’s important to understand how thousands of genes can interact with each other as well as how the related metabolic pathways contribute to plant development and adaptation to the environment (Licciardello et al., 2021). Thus, the identification and characterization of genes controlling agricultural traits and tagging molecular markers constitutes advances for development of new breeding techniques (Soundararajan et al., 2019). In this follow up, the functional genomics in fruit trees has deployed several methodologies to improve the molecular breeding techniques in fruit crops, such as gene expression-based biomarkers, transcriptomic and metabolomics, whole-genome variations and sequence, among others (Licciardello et al., 2021). With the available information, several fruit quality traits can be improved, namely the development of new cultivars with small/larger size, good-flavored fruits, attractive color, sugar and acid levels, reduced juvenile phase, massive and constant yields, reduced susceptibility to fruit cracking, self-compatibility, and improved resistance or tolerance to disease as well as resistance to abiotic stresses like adverse environmental conditions (Soundararajan et al., 2019).

Regarding cracking, it’s known that there are several factors that affect fruit cracking, such as physiological, genetics, environmental and postharvest storage (Khadivi-Khub, 2015; Wang et al., 2021b). Among the several mitigation strategies to prevent cracking, calcium is a key mineral in plant physiology (Winkler and Knoche, 2021). It plays an important role in the pre- and postharvest physiology of most plant and particularly of fruit, being considered a critical nutrient in determining fruit quality (Winkler and Knoche, 2019). For example, calcium application in grape berry appears to induce specific modifications both in skin and in pulp, changing the cuticle structure, playing an important role in preventing cracking (Martins et al., 2020) as well as the inhibition of cell wall disassembly promoting cell wall strengthening and, thus, calcium can prevent grape cracking (Yu et al., 2020). Likewise, the use of calcium to mitigate the risk of pre-harvest rain-cracking of sweet cherry increases the fruit quality, firmness and shelf-life, reducing the cracking susceptibly (Correia et al., 2019; Winkler and Knoche, 2019; Breia et al., 2020; Winkler et al., 2020; Correia et al., 2020a; Matteo et al., 2022). Furthermore, in sweet cherry, the combination of calcium with growth regulators highly reduce the cracking incidence, promoting differential gene expression of some exocarp related genes (Correia et al., 2020b). Thus, a better knowledge about the cuticle related genes can provide new insights about molecular mechanisms involved in cracking of flesh fruits. For example, the understanding of exocarp development in sweet cherry as well as the expression of exocarp-specific genes during fruit growth, maturation, softening, cuticle deposition and sugar transport, can provide great information about their role in conferring cracking resistance (Alkio et al., 2014). Likewise, in watermelon, the identification of genes related to rind hardness and the associated molecular markers can help to understand rind hardness and fruit cracking resistance and, thus, used in future breeding programs, by CRISPR-Cas9 or marker-assisted selection, to create more resistant cultivars (Liao et al., 2020). Moreover, in jujube, the knowledge about the differences in the cuticular wax and the expression of related genes, using cultivars with different susceptibilities to cracking, may provide new insights about prevent cracking (Li et al., 2014), which coupled by possible enhancing or silencing of related genes by gene modification technology, can change the cell wall structure and arrangement, and, thus, help in fruit cracking prevention (Hou et al., 2018).

Actually, the effects of climate changes, like excessive rains, leads to different physiological responses of the crops, affecting fruit growth, development and quality (Hirpo and Gebeyehu, 2019). The fruit ripening, that is, from a green fruit to a ripe fruit, represents a synchronized process with changes in physiological structure and biochemical composition according to interactions among fruits and their environment (García-Gómez et al., 2020). Fruit cracking emerges as a physiological disorder during fruit development as response to genetic or environmental factors (Wang et al., 2021b). The fruit development is controlled by expression of several genes (polygenic expression regulated by hundreds to thousands of genes), with different associated molecular mechanisms. These include biochemical, transcriptional, hormonal or metabolites levels. Thus, when physiological disorders appear, like cracking, the different underlying mechanisms make it challenging to study (García-Gómez et al., 2020). However, in general, an increase of expression of genes related to cell wall mechanisms arises in cracking-resistant cultivars of sweet cherry (Balbontín et al., 2014; Michailidis et al., 2021), apple (Kasai et al., 2008; Joshi et al., 2018) or watermelon (Jiang et al., 2019b). This indicates a common mechanism causing cracking. Likewise, an upregulation of cuticular waxes related genes in apple (Gao et al., 2021; Straube et al., 2021), watermelon (Jiang et al., 2019b) or jujube (Li et al., 2021c) is also correlated with a decrease of cracking index. Moreover, an unbalance of genes related to hormone metabolisms can increase the cracking susceptibility in litchi (Wang et al., 2019a; Wang et al., 2019b; Wang et al., 2021a) and in atemoya (Li et al., 2019). Hou et al. (2022) reported several metabolic pathways involved in cracking jujube fruits, highlighting the complexity of this disorder.

Thus, the combination of genomic, transcriptomic and metabolomic analyses can reveal important knowledge and genetic basis for crops molecular breeding (Zhang and Hao, 2020). Moreover, the understanding about the differences in gene expression during fruit development elucidates the molecular mechanisms of cuticle related genes, playing important roles in preventing or enhancing fruit cracking (Hou et al., 2018).

## Conclusion

Although fruit cracking remains a great challenge to the producers, it is known that the genetic factors play a crucial role in its development. The genetic component makes cracking an attractive field for researchers who work with molecular breeding. The development of different omics technologies, open news perspectives to understand how this disorder occurs at molecular level. The molecular mechanisms involved in cracking are based on correlations as direct proof of concept based on mutations or reverse genetics are still missing. It is known that exocarp-specific transcripts play a crucial role in cracking development, namely genes involved in cuticular membrane cuticular, cell wall mechanisms or cuticular wax biosynthesis. Additionally, by the analysis of the metabolome, which is closely related to phenotype, the regulation of several metabolic pathways can affect the expression of exocarp-specific genes and, consequently, affects the development of fruit cracking. This physiological disorder is enhanced by environmental conditions, such as high temperatures and heavy rain. The scenario of climate change, foreseen for the future, makes it even more urgent to understand the responses of plants to stress (simple or combined), at various levels, especially at the transcriptomic level. Many genes are repressed or induced in stress response, involving a precise regulation of complex stress-gene networks. It is therefore crucial to understand the function of the genes involved, to determine the functional relationships between genes and how they are affected by biotic or abiotic factors. Obtaining a set of candidate genes for molecular breeding programs by genome editing technologies should bring forward crop performance under changing environmental conditions.

## Author contributions

MS, ME-C, BG and MM contributed to the conceptualization of the review. MS wrote the first draft of the manuscript. ME-C, BG and MM supervised, reviewed and edited the manuscript. All authors contributed to manuscript revision, read, and approved the submitted version.
